# Poly-(γ-glutamic acid) Production and Optimization from Agro-Industrial Bioresources as Renewable Substrates by *Bacillus* sp. FBL-2 through Response Surface Methodology

**DOI:** 10.3390/biom9120754

**Published:** 2019-11-20

**Authors:** Da-Young Song, Lebaka Veeranjaneya Reddy, Dimitris Charalampopoulos, Young-Jung Wee

**Affiliations:** 1 Department of Food Science and Technology, Yeungnam University, Gyeongsan Gyeongbuk 38541, Korea; ssongdayoung@daum.net; 2 Department of Microbiology, Yogi Vemana University, Kadapa (A.P.) 516003, India; 3 Department of Food and Nutritional Sciences, University of Reading, Whiteknights, P.O. Box 226, Reading RG6 6AP, UK; d.charalampopoulos@reading.ac.uk

**Keywords:** *Bacillus* sp. FBL-2, optimization, rice bran, poly-(γ-glutamic acid), wheat bran

## Abstract

We optimized culture conditions using *Bacillus* sp. FBL-2 as a poly-(γ-glutamic acid) (PGA) producing strain isolated from cheonggukjang. All experiments were performed under aerobic conditions using a laboratory scale 2.5 L fermentor. We investigated the effects of fermentation parameters (temperature, pH, agitation, and aeration) and medium components (glutamic acid, citric acid, and yeast extract) on poly-(γ-glutamic acid) production, viscosity, and dry cell mass. A non-optimized fermentation method (1.5 vvm, 350 rpm, and 37 °C) yielded PGA, viscosity, and dry cell mass at levels of 100.7 g/L, 483.2 cP, and 3.4 g/L, respectively. L-glutamic acid, citric acid, and yeast extract supplementation enhanced poly-(γ-glutamic acid) production to 175.9 g/L. Additionally, the production of poly-(γ-glutamic acid) from rice bran and wheat bran was assessed using response surface methodology (central composite rotatable design). Agricultural byproducts (rice bran and wheat bran) and H_2_SO_4_ were selected as factors, and experiments were performed by combining various component concentrations to determine optimal component concentrations. Our experimentally-derived optimal parameters included 38.6 g/L of rice bran, 0.42% of H_2_SO_4_, 28.0 g/L of wheat bran, and 0.32% of H_2_SO_4_. Under optimum conditions, rice bran medium facilitated poly-(γ-glutamic acid) production of up to 22.64 g/L, and the use of wheat bran medium yielded up to 14.6 g/L. Based on a validity test using the optimized culture conditions, poly-(γ-glutamic acid) was produced at 47.6 g/L and 36.4 g/L from these respective mediums, and both results were higher than statistically predicted. This study suggests that rice bran can be used as a potential alternative substrate for poly-(γ-glutamic acid) production.

## 1. Introduction

**Figure 1:**
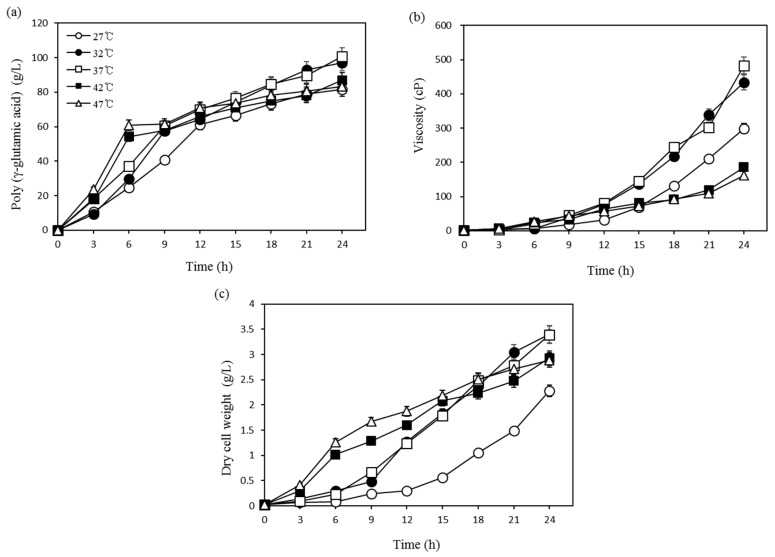
Effects of temperature on (**a**) poly-(γ-glutamic acid) production, (**b**) viscosity, and (**c**) dry cell weight (DCW).

Poly-(γ-glutamic acid) (PGA) is a natural polyaminoic acid and biocompatible compound that is naturally synthesized by members of the genus *Bacillus* during food fermentation. This compound was first discovered by Ivanovics during a study of the film produced by *Bacillus anthracis*. Later, Bovarnick observed the presence of this compound in *Bacillus subtilis* growth medium as a freely secreted polymer [1]. Poly-γ-glutamate is produced as a non-cellular product that is completely biodegradable and soluble in water. Additionally, it is a non-toxic, edible, and environmentally friendly substance that can be separated from food [2]. The molecular structure of poly-(γ-glutamic acid) consists of the repetitive units of D- or L-glutamic acid connected by amide bonds between the α-amino and the carboxyl radicals of the monomer of glutamic acid (Figure 1), and this compound exists as an abnormal anionic homopolypeptide [2]. Poly-(γ-glutamic acid), produced by microorganisms, does not have a fixed molecular weight; however, it typically exists as a macromolecule with a size range of 10 to 1000 kDa [3]. Poly-(γ-glutamic acid) is a polymer that can be used in a wide range of areas, including food, medical, water treatment, cosmetic, and agricultural industries. In the food industry, it can be used as a food supplement, texture enhancer, cryoprotectant, sedimenter, bitter taste reducing agent, hydrogel, bioflocculant, and genealogical feed additive. It also has numerous applications within the health care industry based on its chemical characteristics [4].

The bacteria that produce poly-(γ-glutamic acid) belong to the genus *Bacillus*, and these bacteria include *Bacillus anthracis*, *Bacillus subtilis*, *Bacillus licheniformis*, *Bacillus amyloliquefaciens*, and *Bacillus methylotrophicus*. Each of these microorganisms require different nutritional inputs for the production of poly-(γ-glutamic acid). Based on this, microbes are classified into two groups—glutamate-dependent and glutamate-independent bacteria [2]. The glutamate-dependent bacteria are *B. subtilis* (cheonggukjang), *B. licheniformis* 9945a, *B. subtilis* CGMCC 0833, *B. licheniformis* NK-03, and *B. subtilis* ATCC 15245 (natto). In these bacteria, the yield of poly-(γ-glutamic acid) increases as the concentration of L-glutamic acid is increased within the growth medium [5]. Independent bacteria are more desirable for industrial production than are dependent bacteria due to their low cost and simple fermentation processes. However, the usefulness of these bacteria is limited in scope compared to that of the dependent bacteria [6].

The polymeric materials produced by microorganisms can be used as novel materials in many industries, and therefore, production must occur in large quantities to satisfy industrial demand [7]. One potential problem is that poly (γ-glutamic acid), as a high-molecular material, requires expensive substrates and is restricted in use due to high production costs. To solve this problem, a method is required to produce poly-(γ-glutamic acid) in a more economical manner by replacing expensive substrate with a low-cost substrate. Agricultural byproducts that are disposed as waste and are abundantly available possess sufficient potential as alternative materials for producing poly-(γ-glutamic acid). In recent years, the suitability of agricultural byproducts such as rice straw [8], molasses [9], and dairy compost [10] has been evaluated. Very recently, Anju et al. [11] investigated the comparative production of poly-(γ-glutamic acid) from rice straw, sugar cane waste, sugarcane bagasse, cotton stalks, and sorghum stover.

Rice bran and wheat bran are highly utilized materials due to their distribution of nutrients. Rice bran produced during polishing contains 34–62% carbohydrates, 15–20% fat, 11–15% protein, 7–10% ash, and 7–11% crude fiber, and although it is a byproduct, it accounts for 95% of the nutritional value of rice [12,13]. Approximately 20–30% of the total output of rice is used for maintenance extraction, and the remainder is used for feed or compost. Additionally, wheat bran produced during the milling process of wheat contains 15% protein, 12% starch, 6% ash, and 6% fat, and the majority of the byproducts are used as dietary fiber additives in feed or drinks [14]. The major hurdle in the utilization of these materials as fermentation feedstock is that they cannot be used in their current states by many microorganisms, and instead they require hydrolysis to convert them into simple fermentable sugars. Rice and wheat are among the top three crops worldwide, and their utilization is very low despite the high production of agricultural byproducts [15]. The goal of this study was to determine a means of using these discarded agricultural byproducts for the production of poly-(γ-glutamic acid). To accomplish this, we proposed three objectives—the optimization of fermentation conditions (temperature, pH, agitation, and aeration) using a one-factor-at-a-time method; the improved production of poly (γ-glutamic acid) through repeated batch fermentation by incorporating these optimized fermentation conditions; and the optimization of wheat and rice bran hydrolysis through response surface methodology (RSM) to facilitate the economic production of poly-(γ-glutamic acid).

## 2. Materials and Methods

### 2.1. Bacterial Culture and Inoculum Preparation

In this study, Bacillus sp. FBL-2 KCTC 12962BP was isolated from cheonggukjang, a fermented soybean paste, and was subsequently used for the production of poly-(γ-glutamic acid). Inoculum was prepared from the stock culture by inoculating to the culture medium composed of glucose (10.0 g/L), yeast extract (3.0 g/L), L-glutamic acid (20.0 g/L), KH2PO4 (1.0 g/L), and MgSO_4_·7H_2_O (1.0 g/L, pH 7.0). The inoculum was then incubated at 37 °C on a shaking incubator (Vision Scientific Co., Daejeon, Korea) at 200 rpm for 24 h. To preserve the culture, 50% (v/v) glycerol was added to the culture as a cryoprotectant, and the cultures were stored at −70 °C in a deep freezer until further use.

### 2.2. Poly-(γ-glutamic acid) Production

The bacterial culture described above was used as inoculum (3%) to initiate fermentation. The production medium contained sucrose (5%), L-glutamic acid (10.5%), yeast extract (1.32%), citric acid (1%), KH_2_PO_4_ (0.1%), and MgSO_4_·7H_2_O (0.1%), and this medium was used to study the effect of culture conditions such as temperature, pH, agitation, and aeration by employing a one-factor-at-a-time method. For the substrate optimization studies, rice bran, wheat bran, and the concentration of sulfuric acid were all selected as variable factors, while L-glutamic acid (10.5%), yeast extract (1.32%), citric acid (1%), KH_2_PO_4_ (0.1%), and MgSO_4_·7H_2_O (0.1%) levels were kept constant. All experiments were performed in a 2.5 L fermentor (KF-2.5L; Ko-biotech Co., Incheon, Korea) containing 1 L medium at an incubation period of 24 h. Fed-batch fermentations were performed by periodically supplementing (every 12 h) glutamate and citric acid (50 mL of each substrate at the concentration of 50 g/L and 5 g/L) for 60 h.

### 2.3. Optimization Studies Using RSM

RSM was performed using a central composite design (CCD) to investigate the optimal combination and the key interactions of independent variables in the context of γ-PGA production (Table 1). RSM is a combination of statistical methods that is used for selecting the optimum experimental conditions that require the minimum number of experiments.

$$x_{i}=\frac{X_{i}-X_{0}}{\Delta X_{i}}$$

The experimental variables were coded according to the above equation, where *X_i_* is the actual value of the independent variable, *X_0_* is the independent variable value at the center point, *ΔX_i_* is the step change value, and *x_i_* is the coded value of each independent variable.

As shown in Table 2, to investigate the nature of the response surface in the optimum region, a 2^3^ factorial CCD with eight axial points and six center points was used, and this resulted in a total of 14 experiments. To optimize the production of γ-PGA, the following second-order polynomial equation was used for statistical analysis.

$$y=b_{1}+\sum b_{i}x_{i}\sum b_{ij}x_{i}x_{j}+\sum b_{ii}x_{i}^{2}$$

where *y* is a predicted value, *b*_0_ is a constant, *b_i_*, *b_ii_*, and *b_ij_* are first-order coefficients, second-order coefficients, and interaction coefficients, respectively, *x_i_* is the independent variable of *i*, *x_i_x_j_* is the interaction between independent variables, and *x_i_*^2^ is the second order coefficient. The quality of fit of the model equation was described by the coefficient of determination, *R*^2^, and the significance of statistics was determined using an F-test. The significance of the regression coefficients was investigated by t-test. The computer software used was Design-Expert version 9.0.0 by Stat-Ease, Inc. (USA). The combination of different optimized variables that yielded the maximum response was determined in an attempt to verify the validity of the model.

### 2.4. Analytical Methods

Cell growth was monitored by measuring the optical density using a UV-1600 spectrophotometer (Shimadzu Co., Tokyo, Japan) at 660 nm, and this value was then converted to dry cell weight (DCW, g/L) based on the liner relation of DCW and optical density. The viscosity of the culture broth containing γ-PGA was measured using a DV2T digital rheometer equipped with a spindle CP-42 (Brookfield, Middleboro, MA, USA) at 25 °C and 10 rpm for 30 s. The measured viscosity was corrected using silicon oil standards (44.8 cP and 496 cP at 25 °C). γ-PGA was determined by alcohol precipitation according to the modified method reported by Kunioka and Goto [16]. The fermentation broth was diluted and centrifuged at 32,000× *g* for 30 min. The resulting supernatant was poured into four volumes of cold ethanol. The precipitate was collected and washed with ethanol, and it was then dissolved and dialyzed against deionized water overnight. The dialyzed solution was centrifuged, and the supernatant was lyophilized to prepare pure γ-PGA.

### 2.5. Thin Layer Chromatograph (TLC)

Thin layer chromatography (TLC) was performed to identify hydrolysis products of γ-PGA. Using a 50 mL vial, purified γ-PGA was dissolved in 6 M HCl to a concentration of 1 g/L and hydrolyzed at 110 °C for 1 h. Hydrolysis specimens (2 μL) were added to a silica gel TLC plate (Silica gel 60 F254, 10 × 10 cm) purchased from Merck (Darmstadt, Germany). *n*-Butanol: acetic acid: H_2_O (3:1:1, v/v/v) and 96% ethanol: H_2_O (63:37, v/v) were created for use as the primary developer. After deployment as the primary developer, the deployment of the secondary developer was completed, and a 0.2% ninhydrin acetic acid solution was identified. This solution was dried for 10 min at 110 °C to identify visible hydrolysis products [17].

## 3. Results and Discussion

### 3.1. Optimization Using One-Factor-at-a-Time

To identify the temperature effect and to determine the optimum temperature for γ-PGA production, five experiments were conducted at various temperatures ranging from 27 to 47 °C at 5 °C increments (Figure 1a–c). As shown in the figure, at 37 °C, we observed maximum γ-PGA production (100.7 g/L), viscosity (483.2 cP), and dry cell mass (3.4 g/L), and similar values were obtained at 32 °C (97.1 g/L, 433.1 cP, and 3.4 g/L). Additionally, the production of γ-PGA at 42 °C and 47 °C appeared very rapid at the start of the reaction; however, the final yield and viscosity dropped to low values. This may have been due to rapid cell growth at 42 °C and 47 °C during the early stages and a subsequent lethal effect at the later stages of fermentation. Cheng et al. [18] reported 30 °C as the optimal temperature for *B. licheniformis* A35. Unlike the other studies, Kubota et al. [19] used 42 °C as the optimum temperature for *B. subtilis* F-2-01. In the present study, 37 °C was selected as the optimum temperature for use in further studies.

**Figure 2:**
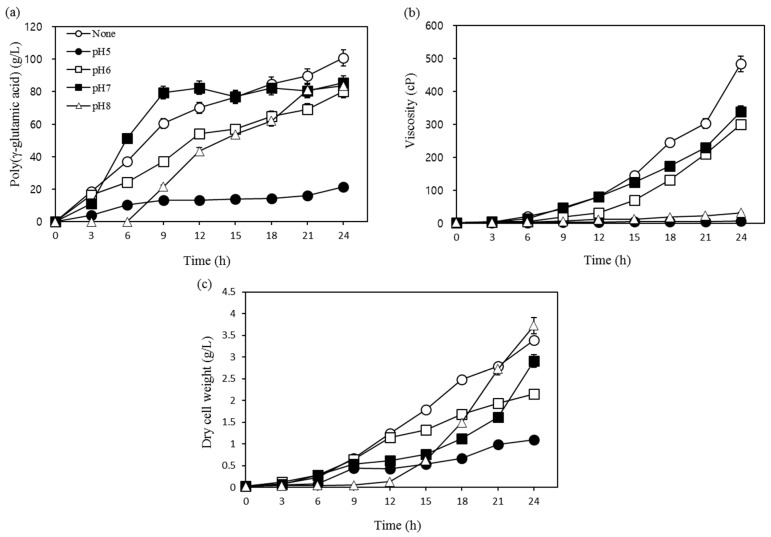
Effects of pH on (**a**) poly-(γ-glutamic acid) production, (**b**) viscosity, and (**c**) dry cell weight.

To determine the optimum pH for γ-PGA production, five experiments were conducted at different initial pH that included control (not adjusted), 5, 6, 7, and 8. As shown in Figure 2a–c, yield, viscosity, and dry cell mass were higher at 100.7 g/L, 483.2 cP, and 3.4 g/L, respectively, at the control pH. These results were similar to those obtained at pH 7, and they were much higher than the results obtained at pH 5 and pH 8 (83.6 g/L). The pH change in the control was not shown in the figure; however, we observed that the initial pH of the control medium was 6.75, and it first decreased to 5.92 and then gradually increased after 18 h. In the pH 5 experiment, the yield of γ-PGA, viscosity, and dry cell mass were significantly reduced, indicating that, in acidic environments, cell growth was impaired and γ-PGA biosynthesis was not accomplished properly. Cromwick et al. [20] reported similar results in regard to pH, where they obtained the highest γ-PGA yield at 6.5, and they suggested that, at this pH, the molecular weight and the substrate consumption were also increased. Zanuy et al. [21] demonstrated that the carboxyl group of γ-PGA possessed different structures of exposed anionic polymers depending on pH changes within the solution. Therefore, it is assumed that structural changes within the polymer that result from changes in the pH will affect the viscosity. Our results suggest that there is no need to adjust the pH of the medium to allow for improved γ-PGA synthesis.

**Figure 3:**
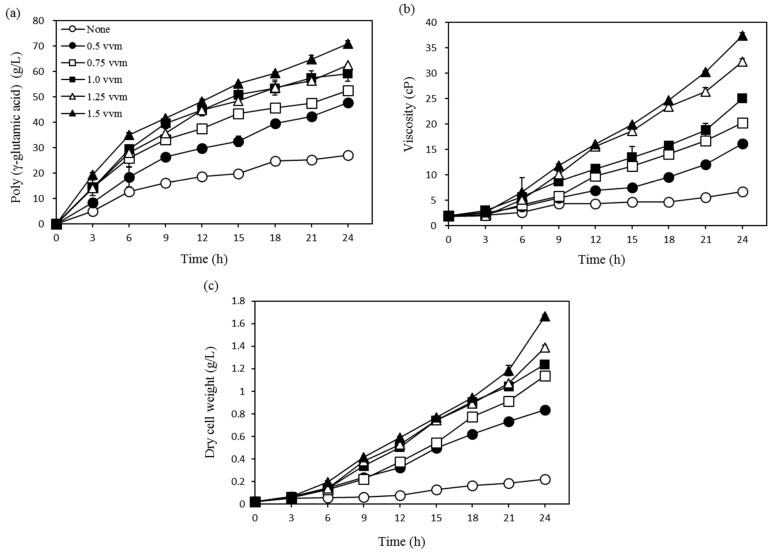
Effects of aeration rate on (**a**) poly-(γ-glutamic acid) production, (**b**) viscosity, and (**c**) dry cell weight.

To determine the effect of aeration rate on γ-PGA production, we utilized *Bacillus* sp. FBL-2. This experiment was conducted using six variables that included control (no air), 0.25 vvm, 0.5 vvm, 0.75 vvm, 1.00 vvm, 1.25 vvm, and 1.5 vvm, and it was performed using a 2.5 L fermentor under constant temperature and agitation (37 °C and 200 rpm, respectively) (Figure 3a–c). A high γ-PGA yield (70.9 g/L), viscosity (39.9 cP), and dry cell mass (1.6 g/L) were observed under conditions of 1.5 vvm air sparging, and these values were 2.8 times higher than those obtained under control conditions, where γ-PGA yield, viscosity, and dry cell mass were 27.1 g/L, 6.7 cP, and 0.2 g/L, respectively. We observed that yield, viscosity, and dry cell mass all gradually increased as the aeration rate increased from 0.5 vvm to 1.5 vvm. It is likely that the high aeration promoted cell growth to improve the production of γ-PGA. Additionally, studies by Cromwick [20] and Bajaj and Singhal [22] reported that increased oxygen supply improved cell growth and ultimately doubled the maximum dried cell mass while depleting carbon sources more rapidly to provide enhanced production of γ-PGA. The results of this study confirmed that increased oxygen supply improved production yields, highlighting the role of oxygen supply as an important factor in the production of γ-PGA. Additionally, these results confirmed that a 1.5 vvm aeration rate is optimal for γ-PGA fermentation.

**Figure 4:**
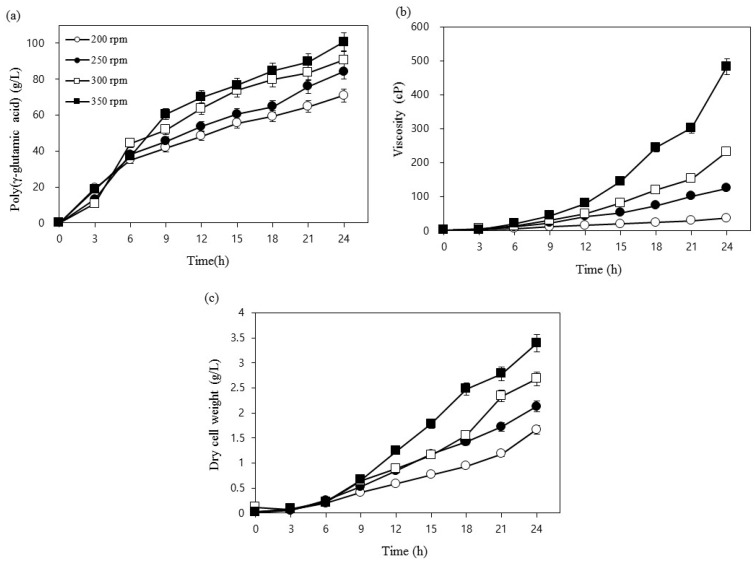
Effects of agitation speed on (**a**) poly (γ-glutamic acid) production, (**b**) viscosity, (**c**) dry cell weight.

To determine the optimum agitation speed for the production of γ-PGA by *Bacillus* sp. FBL-2, four variable speeds (200, 250, 300, and 350 rpm) were screened (Figure 4a–c). For all the tested agitation speeds, the highest γ-PGA production (100.7 g/L), viscosity (483.2 cP), and dry cell mass (3.4 g/L) were obtained at 350 rpm, and the lowest γ-PGA production yields (70.9 g/L), viscosity (37.5 cP), and dry cell masses (1.7 g/L) were observed at 200 rpm. We observed that, as the viscosity increased exponentially, the amount of dry cell mass and the γ-PGA yield also increased proportionally as the stirrer speed was increased. Our results are in agreement with those of previous investigations that indicated that increased agitation resulted in enhanced γ-PGA production [22]. According to the studies by Cromwick et al. [20] and Bajaj and Singhal [22], a higher viscosity within the fermented solution resulted in a decrease in the efficiency of oxygen transmission, ultimately affecting the transfer of nutrients and the distribution of air and oxygen. Therefore, it is likely that increasing the stirring speed resulted in a more even oxygen supply, ultimately increasing cell growth, viscosity, and the production of γ-PGA. Based on the results of this study, it was determined that the stirring speed is an essential parameter for the production of γ-PGA, and we selected agitation at 350 rpm for further studies.

### 3.2. Fed-Batch Fermentation

**Figure 5:**
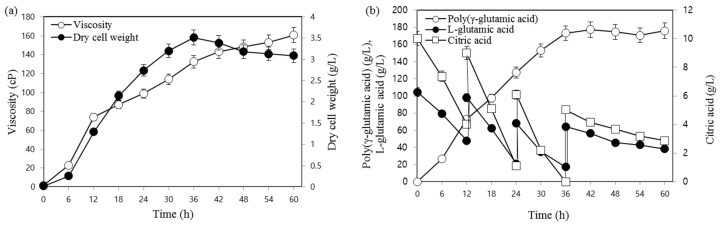
Poly-(γ-glutamic acid) production following the addition of L-glutamic acid, citric acid, and yeast extract. (**a**) Viscosity and dry cell weight; (**b**) poly(γ-glutamic acid) production and consumption of L-glutamic acid and citric acid.

To overcome the shortcomings of batch fermentation that result from such events as nutrient depletion due to microbial metabolism, fed-batch fermentation was conducted under previously optimized culture conditions. Based on the results of the preliminary experiments, substrates were added every 12 h, and the fermentation time was increased up to 60 h. Samples were collected every 6 h to assess the amount of γ-PGA and the residual substrate concentration (Figure 5). As shown in the figure, a maximum γ-PGA production of 175.9 g/L was achieved after 60 h of incubation, and 38.3 g/L and 2.87 g/L of L-glutamic acid and citric acid, respectively, were unused and existed as residuals. L-glutamic acid and citric acid are important precursors for the production of γ-PGA; however, it is assumed that the addition of very high substrate concentrations inhibits cell growth at or above the concentration limit and that the length of the molecular chain of γ-PGA is dependent upon the substrate concentration, as short chain lengths result in low viscosity. Poly-(γ-glutamic acid) production obtained from each optimization step is presented in Table 3. As indicated in the table, the resulting concentration of γ-PGA in the present study was very high compared to that detailed in previous reports. Yoon et al. [23] achieved only 35 g/L of γ-PGA with 1g/L/h productivity using optimized fed-batch fermentation by *Bacillus licheniformis*. Jeong et al. [24] and Zhang et al. [9] also reported similar low production of γ-PGA using *Bacillus subtilis* RKY3 and an optimized fermentation process.

### 3.3. γ-PGA Production Using Agricultural Byproducts

For the production of γ-PGA using agricultural byproducts, these byproducts must first be hydrolyzed into simple fermentable sugars. Rice bran [composition in percentages (%): moisture, 5.4; fiber, 30.17; carbohydrate, 52.5; protein, 17.5; ash, 5] and wheat bran [composition in percentages (%): moisture, 8.4; fiber, 33.5; carbohydrate, 62.5; protein, 12.5; ash, 6] were selected as agricultural byproducts and hydrolyzed using dilute H_2_SO_4_. To determine the optimal concentrations of rice/wheat bran and H_2_SO_4_, a central composite design method was used for a total of 14 experiments that included six center points to estimate the error of the experiment. The experimental results were then derived through the use of multiple regression analyses (Table 4a and b). A second-order polynomial equation was obtained to predict the production of γ-PGA.

*y* = −2.74475 + 0.979296 *x*_1_ + 32.09556 *x*_2_ − 0.014965 *x*_1_*x*_2_ − 0.01282 *x*_1_^2^ − 39.8736 *x*_2_^2^

In the above polynomial equation, y represents the expected yield of γ-PGA (g/L), *x*_1_ represents rice bran, and *x*_2_ is the coded value of H_2_SO_4_.

*y* = −3.11752 + 0.978556 *x*_1_ + 24.6798 *x*_2_ − 0.14245 *x*_1_*x*_2_ − 0.01664 *x*_1_^2^ − 31.823 *x*_2_^2^

In the above polynomial equation, y represents the expected production of γ-PGA (g/L), *x*_1_ represents the wheat bran, and *x*_2_ is the coded value of H_2_SO_4_.

The results of the ANOVA analysis indicated that the *F*-value of the regression formula in the experimental model was statistically significant, as the *p*-value was less than 0.0001 for rice bran and wheat bran (37.5664 and 29.51582, respectively) (Table 4a and b). The coefficients of determination for this model were 0.9591 and 0.9485, respectively, indicating that the regression formula is highly useful. The predicted values for the production of γ-PGA according to this model formula were 95.91% and 94.85%, respectively. Based on this, the secondary polynomial obtained by multiple regression analyses could be characterized as possessing a high level of confidence, and the level at which γ-PGA production was predicted was within the range of each variable.

**Figure 6:**
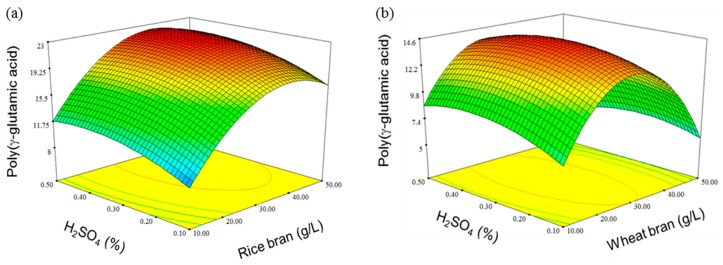
Response surface and contour plots of poly-(γ-glutamic acid) production by *Bacillus* sp. FBL-2. (**a**) rice bran; (**b**) wheat bran.

Based on the regression equation, the three-dimensional response surface graph visualizes the interaction of important factors and the correlation between the production volume of γ-PGA and the concentration. As shown in Figure 6, the two response surface curves exhibited a convex shape and outlined the position of the optimum conditions. The optimal levels for each factor as calculated by the regression formula were 38.6 g/L and 0.42% H_2_SO_4_ for rice bran and 28.0 g/L and 0.32% H_2_SO_4_ for wheat bran. For the predicted response at the optimum level, γ-PGA production was established using centralization planning experiments that incorporated levels of rice bran and wheat bran at 22.64 g/L and 14.6 g/L, respectively. γ-PGA synthesis was improved five-fold by RSM using *Bacillus licheniformis* NCIM 2324 [25]. RSM provides an effective means for optimizing PGA production.

### 3.4. Verification of the Optimal Model

The optimization model was verified by experimenting with optimized conditions of two factors important for the production of γ-PGA. *Bacillus* sp. FBL-2 was inoculated with rice bran or wheat bran (38.6 g/L, 0.42% H_2_SO_4_ and 28 g/L, 0.32% H_2_SO_4_) under previously optimized culture conditions. Rice bran medium produced higher γ-PGA (47.6 g/L), viscosity (21.4 cP), and dry cell mass (0.46 g/L) compared to those produced using the wheat bran medium [γ-PGA (36.4 g/L), viscosity (8.2 cP), and dry cell mass (0.44 g/L)]. The yields of γ-PGA from rice bran and wheat bran as predicted by the regression formula were 22.64 g/L and 14.6 g/L, respectively. The experimental values, however, were 2.1 times higher than the predicted yields. In the present study, the possibility of utilizing economically cheap substitutes in place of the expensive medium components was explored. For example, simply replacing sucrose with rice bran could lower production costs on average by a factor of 10. Similarly, the replacement of the more commonly used and expensive precursors with the more inexpensive and available substitutes would also result in a considerable reduction in production costs. Therefore, the results of our experiments suggest that agricultural byproducts such as rice bran and wheat bran could provide suitable substitutes for the effective economization of γ-PGA production.

### 3.5. Analysis of Hydrolysis Products of γ-PGA

**Figure 7:**
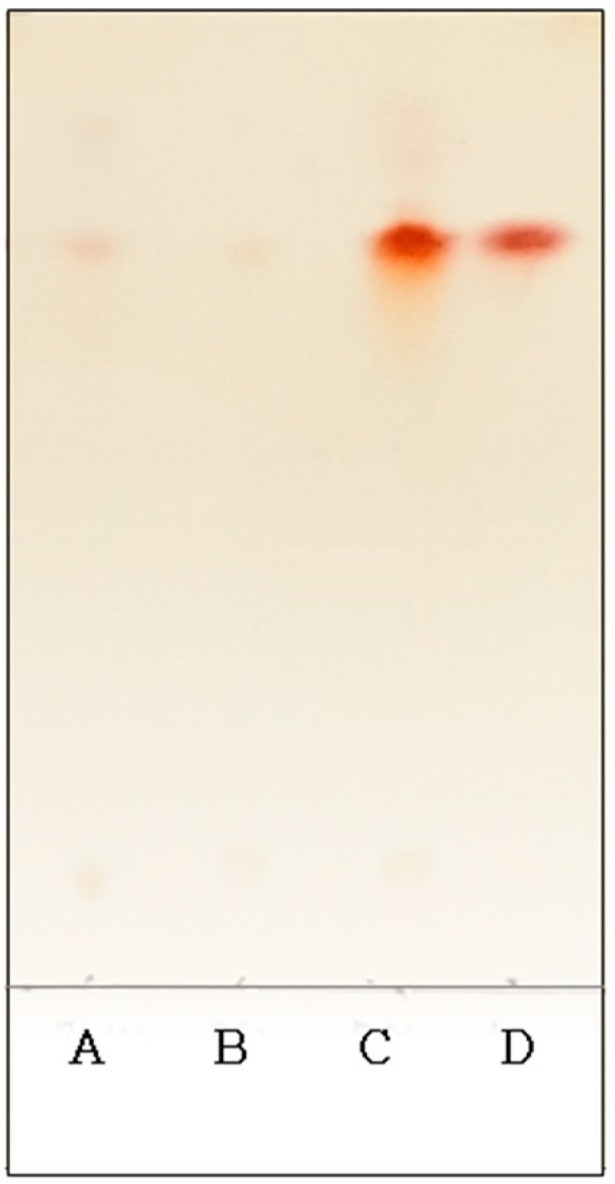
Thin layer chromatography of poly-(γ-glutamic acid) hydrolysate. Lane A, precipitated poly-(γ-glutamic acid); Lane B, after addition of HCl on poly-(γ-glutamic acid); Lane C, after addition of HCl on poly-(γ-glutamic acid) and heat treatment at 110 °C for 30 min; Lane D: glutamic acid standard.

Thin layer chromatography (TLC) was used to verify the hydrolysis of viscous polymers produced under optimal conditions. The peptides that comprise γ-PGA are connected in a γ-combination. After hydrolysis, an analysis of γ-PGA hydrolysis products revealed that the R_f_ value of the hydrolysis products and the R_f_ value of glutamic acid were indicative of the presence of a viscous polymer that was identified as γ-PGA, a polyamino acid composed of glutamic acid (Figure 7).

## Figures and Tables

**Table 1:** Variables and experimental design levels used in the central composite design for rice bran and wheat bran.

Independent Variables	Coded Symbols	Levels				
		−1.414	−1	0	1	1.414
Bran (g/L)	*X_1_*	1.7	10	30	50	58.3
H_2_SO_4_ (%, v/v)	*X_2_*	0.017	0.1	0.3	0.5	0.583

**Table 2:** Central composite design (CCD) of two independent variables.

Run No.	Coded Variable Level	
	*X*_1_	*X*_2_
1	−1	−1
2	1	−1
3	−1	1
4	1	1
5	−1.414	0
6	1.414	0
7	0	−1.414
8	0	1.414
9	0	0
10	0	0
11	0	0
12	0	0
13	0	0
14	0	0

**Table 3:** Poly-(γ-glutamic acid) production obtained from each optimization step.

Optimization Step	Dry Cell Weight (g/L)	Viscosity (cP)	Poly-(γ-glutamic acid) Production (g/L)	Poly-(γ-glutamic acid) Production (Fold)
Before optimization *	0.2	6.7	27.1	1.0
After fermentor optimization **	3.4	483.2	100.7	3.7
After fed-batch fermentation ***	3.1	160.9	175.9	6.5

*: The fermentation was performed using a 2.5 L fermentor containing 1 L of fermentation medium at 200 rpm and 37 °C. **: The fermentation was performed using a 2.5 L fermentor containing 1 L of fermentation medium at 1.5 vvm, 350 rpm, and 37 °C. ***: The fermentation was performed using a 2.5 L fermentor containing 1 L of fermentation medium at 1.5 vvm, 350 rpm, and 37 °C.

**Table 4:** Analysis of variance (ANOVA) for the response surface quadratic model for poly-(γ-glutamic acid) production by *Bacillus* sp. FBL-2. (**a**) Rice bran; (**b**) wheat bran.

	Source	Sum of Squares	Degree of Freedom	Mean Square	*F*-Value	*p*-Value Prob > *F*
(**a**)	Model	375.7881	5	75.15762	37.56641	< 0.0001
	Residual	16.00528	8	2.00066		
	Lack of Fit	13.1064	3	4.3688	7.53532	0.0265
	Pure Error	2.898882	5	0.579776		
	Corrected Total	391.7934	13			
	*R*^2^ = 0.9591; adjusted *R*^2^ = 0.9336; adequately precision = 17.625; CV = 8.11%.					
(**b**)	Model	346.0666	5	69.213397	29.51582	<0.0001
	Residual	18.75965	8	2.344356691		
	Lack of Fit	17.97731	3	5.992436649	38.29799	0.0007
	Pure Error	0.782344	5	0.156468716		
	Corrected Total	364.8563	13			
	*R*^2^ = 0.9485; adjusted *R*^2^ = 0.9164; adequately precision = 15.053; CV = 15.33%.					
